# Retinoic acid induced specific changes in the phosphoproteome of C17.2 neural stem cells

**DOI:** 10.1111/jcmm.18205

**Published:** 2024-03-20

**Authors:** Cheng Zhang, Lite Ge, Huali Xie, Xiaoqian Liu, Chengfeng Xun, Yan Chen, Haiyan Chen, Ming Lu, Ping Chen

**Affiliations:** ^1^ The National & Local Joint Engineering Laboratory of Animal Peptide Drug Development, College of Life Sciences Hunan Normal University Changsha PR China; ^2^ Guangdong Provincial Key Laboratory of Biotechnology for Plant Development, School of Life Science South China Normal University Guangzhou PR China; ^3^ Hunan Provincial Key Laboratory of Neurorestoratology, the Second Affiliated Hospital Hunan Normal University Changsha PR China; ^4^ Department of Neurology, Second Xiangya Hospital Central South University Changsha PR China

**Keywords:** differentiation, NSCs, phosphoproteome, proliferation, retinoic acid

## Abstract

Retinoic acid (RA), a vitamin A derivative, is an effective cell differentiating factor which plays critical roles in neuronal differentiation induction and the production of neurotransmitters in neurons. However, the specific changes in phosphorylation levels and downstream signalling pathways associated with RA remain unclear. This study employed qualitative and quantitative phosphoproteomics approaches based on mass spectrometry to investigate the phosphorylation changes induced by RA in C17.2 neural stem cells (NSCs). Dimethyl labelling, in conjunction with TiO_2_ phosphopeptide enrichment, was utilized to profile the phosphoproteome of self‐renewing and RA‐induced differentiated cells in C17.2 NSCs. The results of our study revealed that, qualitatively, 230 and 14 phosphoproteins were exclusively identified in the self‐renewal and RA‐induced groups respectively. Quantitatively, we successfully identified and quantified 177 unique phosphoproteins, among which 70 exhibited differential phosphorylation levels. Analysis of conserved phosphorylation motifs demonstrated enrichment of motifs corresponding to cyclin‐dependent kinase and MAPK in the RA‐induced group. Additionally, through a comprehensive literature and database survey, we found that the differentially expressed proteins were associated with the Wnt/β‐catenin and Hippo signalling pathways. This work sheds light on the changes in phosphorylation levels induced by RA in C17.2 NSCs, thereby expanding our understanding of the molecular mechanisms underlying RA‐induced neuronal differentiation.

## 1 INTRODUCTION

Neural stem cells (NSCs) are self‐renewing multipotent cells that play crucial roles in early nervous system development, as well as postnatal regeneration and repair of nervous tissue. These cells possess the potentials to generate differentiated neural cells in central nervous system, including neurons, astrocytes, and oligodendrocytes.^1^, ^2^, ^3^ Nervous system diseases are intractable problems that can cause function losing of sensation, motor and memory with unclear pathogenesis. Traditional drug treatments only offer temporary relief and fail to restore function, necessitating the exploration of new therapeutic approaches. Recent studies have reported that cell therapy based on NSC has potential to treat nervous system diseases through regeneration of neural cells. Retinoic acid (RA), a potent cell differentiating factor, regulates the proliferation and differentiation of NSCs, making it a promising therapeutic agent for nervous system diseases.

RA is an effective cell differentiating factor which is derived from vitamin A by two specific alcohol and aldehyde dehydrogenases catalysing.^4^ The liver and the brain have the function of RA synthesis, especially the liver is the main site of this compound synthesis. Thus, RA in the brain may originate both from endogenous and exogenous synthesis.^5^ RA signal is transduced by retinoic acid receptor (RAR) and the retinoid X receptor (RXR), which are members of the nuclear receptor super‐family of ligand‐activated transcription factors.^5^ There are three kinds of RAR (RARα, RARβ and RARγ) that RA can bind with, and form three different kinds of heterodimer complexes with RXR (RXRα, RXRβ and RXRγ). Each of RXR may interact with various sets of retinoid response elements regulating expression of different arrays of genes.^6^, ^7^ Many downstream effectors of RAR signalling, such as p27Kip1, p21 and CDK, are considered potential cell cycle regulators.^8^, ^9^, ^10^, ^11^ In addition, RA is involved in maintaining the differentiated state of adult neurons. Disruption of this signalling is associated with motor neuron degeneration (motor neuron disease), Alzheimer's disease development and Parkinson's disease development.^12^, ^13^, ^14^ RA also regulate the expression of neurogenesis genes, such as brain‐derived neurotrophic factor (BDNF) and neuronal growth cone guidance,^15^ and the cell cycle by halting proliferation. Due to its capacities, RA is a clinically established drug for chemoprevention and differentiation therapy of some cancers such as acute promyelocytic leukaemia. Moreover, in brain cells, RA‐induced regulatory and adaptive reactions include increased expression of different neuronal transmitter phenotypes, maturation/differentiation of stem cells. Previous studies showed that RA is involved in the switch between proliferation and differentiation of various stem/progenitor cells like embryonic stem cells and neuroblastoma cells.^16^, ^17^, ^18^ Overall, these studies suggested that RA could be used as a therapeutic molecule for the induction of axon regeneration and the treatment of neurodegeneration.^19^, ^20^, ^21^

The C17.2 NSCs are mouse‐derived multipotent avian myelocytomatosis viral‐related oncogene immortalized neural stem cells isolated from mouse cerebellum. It is a prototypical and stable NSC line that has been successfully used in in vitro studies of neural cell activity. Several studies have used it as a model for studying NSC differentiation and proliferation and the effects of various signalling pathways on NSC behaviour. The C17.2 NSC line is also a powerful tool for screening compounds for potential therapeutic applications.^22^, ^23^, ^24^ Moreover, C17.2 cells have also served as a model for proteomics studies.^25^, ^26^, ^27^ They can also develop into neuronal or glial phenotypes, depending on the induced conditions.^28^ Overall, the C17.2 NSC line is an essential tool for neural cell activity, differentiation and proliferation. Therefore, we selected C17.2 cells as the model for research and analysis.

Reversible protein phosphorylation plays a critical role in numerous cellular signalling pathways and biological processes in NSCs, such as Wnt/β‐catenin signalling pathway and cell cycle progression. Currently, mass spectrometry (MS)‐based phosphoproteomics has become a powerful technology with its superiority in phosphopeptide identification as well as quantification and phosphosite localization at the system‐wide level. Previous study investigated the phosphoproteomic of RA‐induced NSCs in early stage.^29^ However, the mechanism of RA‐induced neuronal differentiation in the late stage is still unclear. In this study, to further understand the mechanism of RA‐induced neuronal differentiation and the RA‐induced cellular signalling networks in late stage of NSCs, we performed comprehensive qualitative and quantitative phosphoproteomics analysis of C17.2 NSCs during RA treatment in 7 days. C17.2 NSCs are mouse‐derived multipotent avian myelocytomatosis viral‐related oncogene immortalized neural stem cells isolated from mouse cerebellum. They can develop into neuronal or glial phenotypes, depending on the induced conditions.^28^ Our results might provide a valuable resource of phosphorylation proteins and sites information for NSC self‐proliferation and RA‐induced differentiation. Additionally, they will shed light on functional analysis of phosphorylation in NSC self‐proliferation and RA‐induced differentiation.

## 2 MATERIALS AND METHODS

### 2.1 Cell culture and RA treatment

The C17.2 NSCs were derived from the outer granular layer of the cerebellum of a 4‐day‐old newborn mouse. It was immortalized by retroviral transfection with the c‐myc oncogene and stably transfected with the lacZ gene.^28^ The C17.2 NSCs were maintained in Dulbecco's modified Eagle's medium (DMEM) supplemented with 10% fetal bovine serum, 5% horse serum and 2 mM glutamine in a humidified atmosphere of 5% CO_2_, 95% air at 37°C. After plating for 24 h, the cells were divided to control group and treatment group. In treatment group, the cells (about 50%–60% confluency) were treated with 1 μM RA to induce differentiation. The medium containing fresh RA was replaced every 2 days, and after 7 days, the cells were harvested for further analysis.

### 2.2 Immunofluorescence analysis

The cells were fixed with 4% paraformaldehyde, and permeabilized with 0.5% Triton X‐100 in PBS. To minimize non‐specific binding, the cells were then blocked with a blocking buffer containing 5% goat serum in PBS, followed by incubation at 4°C for 30 min. Subsequently, the cells were incubated with the primary antibody at 4°C for 2 h, after removing the blocking buffer. After incubation, the cells were washed with PBS and incubated in the dark room with a secondary antibody at a 1:250 dilution for 1 h. Following another wash with PBS, the nuclei were stained with Hoechst 33342 (1:1000, Invitrogen‐Molecular Probes) at dark room for 3 min. The cover slips were mounted in glycerol on microscope slides and sealed with nail polish. Images were acquired using a fluorescence microscope. The primary antibodies included anti‐β‐III tubulin (Tuj‐1) (mouse, 1:2000, Sigma Aldrich) and nestin (mouse, 1:500, Santa Cruz Biotechnology). The secondary antibodies used CoraLite594‐conjugated Goat Anti‐Mouse (1:200; Proteintech). Immunopositive cells were observed under a fluorescence microscope (IX71, Olympus). We calculated differentiation ratios for RA induced after 7 days of differentiation using Image‐Pro Plus 6.0 software (Media Cybernetics, Silver Spring) by counting the number of cells stained with DAPI (total cells) and those marked by specific markers; 10 random fields from three independent experiments (*n* = 5) were counted. An immunopositive ratio was determined by dividing the number of immunopositive cells (nestin or Tuj‐1) by the total number of cells (DAPI).

### 2.3 Protein extraction

Cells with or without RA treated (Self‐renewal group and RA‐induced group) were homogenized in cell extraction buffer (2% SDS, 0.1 mM DTT in 0.1 M Tris–HCl pH 7.6) and lysed by 20 s sonication. Subsequently, the lysates were cleared by centrifugation at 16,000 × *g* for 10 min at 4°C. The supernatant was transported and protein concentrations were measured using a BCA method.

### 2.4 Filter‐aided sample preparation digestion

Filter‐aided sample preparation was used for digestion following the protein extraction as the described in the protocol by Wisniewski with some modifications.^30^ Briefly, 200 μg protein samples were diluted with eightfold volume of 6 M urea in 0.1 M Tris–HCl (pH 8.5) in the filter unit. Then, filter unit were centrifugated at 14,000 × *g* for 15 min and flow‐through form the collection tube was discarded. After that, the samples were reduced with 10 mM dithiothreitol (DTT) at 37°C for 1 h, alkylated with iodoacetamide in the dark for 45 min. The samples were washed with 6 M urea in 0.1 M Tris–HCl (pH 8.0) and 25 mM NH_4_HCO_3_. Then, the samples were digested by trypsin with an enzyme‐to‐protein ratio of 1/40 (w/w) at 37°C for 14 h. The digestion solution was collected by 14,000 × *g* centrifugation in 10 min. Then, sample were dried in speed‐vacuum concentrator (savant DNA 120, thermo scientific) and stored at −20°C for further analysis.

### 2.5 Isotopomeric dimethyl labelling and fractionation

The derived peptides from the different samples were labelled using isotopomeric dimethyl labels, following a previously described method with some modifications.^31^ In brief, two C18 SepPak column were washed twice with ACN and solvent A (2% ACN and 0.1% formic acid). Each of the different samples were separately loaded into a SepPak column and washed with 2 mL of solvent A. Then, the SepPak columns were flushed five times with 1 mL of the respective labelling reagent (light or heavy). Following this, 2 mL of solvent A was used to wash the SepPak columns, and then the labelled samples were eluted and collected from the SepPak columns with 500 mL of solvent B (80% ACN and 0.1% formic acid). The labelled samples were mixed and dried in speed‐vacuum concentrator. Dried samples were subjected to the first dimensional fractionation procedure on high‐pH reverse chromatography column (Agilent, ZORBAX Extended‐C18 2.1). The mobile phase consisted of buffer A (10 mM ammonium formate dissolved in 5% ACN, pH = 10.0) and buffer B (10 mM ammonium formate dissolved in 90% ACN, pH = 10.0). The elution gradient was from 5% to 30% buffer B linearly over 40 min with a 0.3 mL min^−1^ flow rate. A total of eight fractions were collected and prepared for the following LC–MS analysis.

### 2.6 Phosphopeptides enrichment

Phosphopeptide enrichment was performed as described previously.^32^ TiO_2_ beads (Sachtopore‐NP, 20 μm, 300 Å; ZirChrom) were employed for the enrichment of phosphopeptides. Initially, peptides were incubated with TiO_2_ beads at a ratio of 1:10 (w/w) in a loading buffer composed of 60% ACN, 2% TFA and glutamic acid saturation. Subsequently, the TiO_2_ beads were sequentially washed with washing buffer 1 (50% ACN, 0.5% TFA) and washing buffer 2 (50% ACN, 0.1% TFA). Finally, the bound phosphopeptides were eluted using 10% NH_4_OH, and the eluates were combined and dried using a vacuum concentrator.

### 2.7 Mass spectrometric analysis

The reverse phase nano‐LC–MS/MS analysis was performed on the Eksigent nanoLC‐Ultra™ 2D System (AB SCIEX, Concord, ON). The lyophilized fractions were suspended in 2% acetonitrile containing 0.1% formic acid, and loaded on ChromXP C18 (3 μm, 120 Å) nanoLC trap column. The mobile phase solvents were composed of water/acetonitrile/formic acid (solvents A, 98/2/0.1%; solvents B, 2/98/0.1%). Firstly, desalting procedure were carried out at 2 μL/min for 10 min with 100% solvent A. Then, an elution gradient of 5%–40% acetonitrile (0.1% formic acid) in 70 min was employed on an analytical column (75 μm × 15 cm, C18, 3 μm, 120 Å, ChromXP Eksigent). LC–MS/MS analysis was performed on a Triple TOF 5600 System (AB SCIEX, Concord, ON) fitted with a Nanospray III source (AB SCIEX, Concord, ON). Data were acquired using an ion spray voltage of 2.4 kV, curtain gas of 30 PSI, nebulizer gas of 5 PSI and an interface heater temperature of 150°C. The MS was operated with TOF‐MS scans ranges from 400 to 1250 m/z. For IDA, survey scans start from 100 to 1500 m/z and were acquired in 250 ms and as many as 30 product ion scans (80 ms) were collected if exceeding a threshold of 200 counts/s and with a +2 to +5 charge‐state. A Rolling collision energy setting was applied to all precursor ions for collision‐induced dissociation. Dynamic exclusion was set for 1/2 of peak width (about 16 s).

### 2.8 Western blotting

The C17.2 NSCs treated with or without RA were lysed on ice using RIPA buffer containing protease inhibitors (P8465, Sigma). The lysates were then quantified using a BCA protein assay kit (Pierce). Equal amounts of total protein (10 μg) were separated by electrophoresis on 12% Bis‐Tris gels and transferred onto a PVDF (polyvinylidene fluoride) membrane (FFP24, Beyotime, China). The membrane was subsequently probed with primary antibodies overnight at 4°C. The primary antibodies used were as follows: anti‐AKT (1:2000, 10,176‐2‐AP, Proteintech, USA), anti‐p‐AKT (1:2000, 66,444‐1‐Ig, Proteintech), anti‐β‐actin (1:5000, 66,009‐1‐Ig, Proteintech), anti‐GFAP (1:10000, ab68428, Abcam), anti‐β‐III tubulin (Tuj‐1) (mouse, 1:2000, ab18207, Abcam) and nestin (mouse, 1:500, Proteintech). The secondary antibodies used Goat anti‐Mouse IgG (H + L) Secondary Antibody (1:5000, AWS0001, Abiowell) And Goat anti‐Rabbit IgG (H + L) Secondary Antibody (1:5000, AWS0002, Abiowell). After washing, the membrane was incubated with a peroxidase‐conjugated secondary antibody for 2 h at room temperature. Immunoreactive bands were detected using an ECL kit following the manufacturer's instructions. Subsequently, the membrane was reprobed with anti‐β‐actin as an internal loading control.

### 2.9 Data analysis

The MS/MS data were analysed for protein identification and quantification using ProteinPilot Software v.4.5 (Sciex Inc.). The global false discovery rate was estimated with the integrated PSPEP tool in the ProteinPilot Software to be 1.0% after searching against a decoy concatenated *Mus musculus* protein database containing 51,459 entries. The database search parameters were set as the followings: Isotopomeric dimethyl peptide‐labelling quantification, oxidation (M), acetyl (Protein‐N term) and phospho (S/T/Y) was set as variable modifications, phosphorylation emphasize, cysteine modified with iodoacetamide, trypsin digestion, MS and MS/MS tolerance of 20 ppm and 0.1 Da, respectively, thorough searching mode, minimum threshold of 95% confidence (unused protein score >1.3) at protein level. Proteins with a fold change of more than 1.5 or less than 0.67 were determined as differentially expressed. All experiments were repeated at least three times, with the results expressed as the mean ± standard deviation (SD) unless stated otherwise.

Motif enrichment analysis was conducted using Motif‐X.^33^ The six amino acids upstream and downstream of the phosphorylation sites were extracted from the protein sequences and submitted to Motif‐X with the following parameter setting: a minimum occurrence of 20 and a significance of 10^−6^. Heat maps were visualized using R Packages pheatmap (https://cran.rstudio.com/web/packages/pheatmap/index.html). Fold enrichments of specific GO functions were obtained using the DAVID database^34^ and was visualized using R packages ggplot2 (https://ggplot2.tidyverse.org). Fold enrichments of Pathway were obtained using KEGG database.^35^ The protein–protein interaction network was generated by STRING^36^ with the following parameter setting: a medium confidence score of 0.4, and interactions were presented based on textmining, experiments and databases. The protein–protein interaction results of STRING analysis were visualized using Cytoscape version 3.4.0.

## 3 RESULTS

### 3.1 RA induce differentiation of C17.2 NSCs into neuron

**FIGURE 1:**
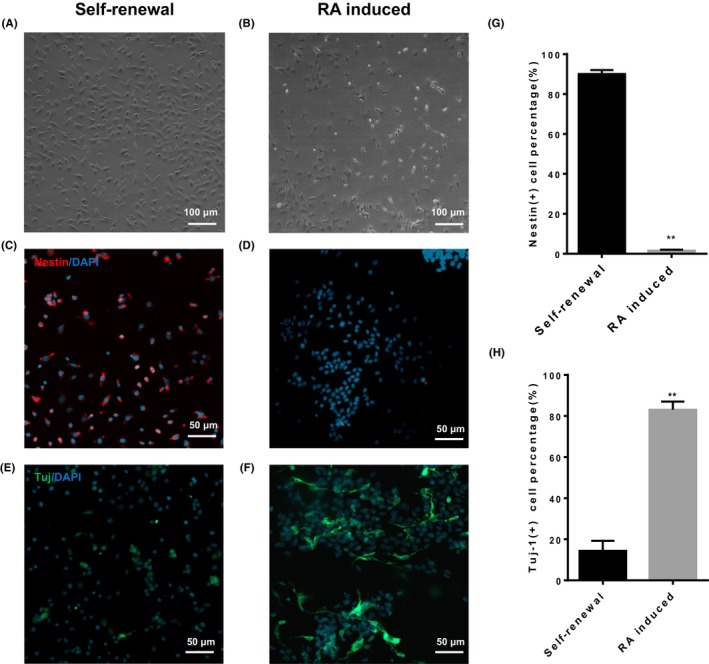
Differentiation of C17.2 NSCs at RA treatment. (A, B) Representative phase contrast photomicrographs demonstrating phenotypic differences between neuritogenesis in self‐renewal group and RA‐induced group for 7 days, Scale bar:100 μm; (C, D) Nestin signals in self‐renewal group and RA‐induced group, Red signal was from staining of neural stem cell marker nestin. Nuclei were counterstained with Hoechst 33342, Scale bar:50 μm; (E, F) Tuj‐1 signals in self‐renewal group and RA‐induced group, green signal was from staining of neuron‐specific marker Tuj‐1. Nuclei were counterstained with Hoechst 33342, Scale bar: 50 μm. (G, H) Nestin^+^ and Tuj‐1^+^ quantification, compared with the self‐renewal group. *n* = 3 per group, **p< 0.01.

To investigate the role of RA in differentiation of C17.2 NSCs into neuron, the morphology of NSCs with or without RA treatment was observed. After cultured in the presence of RA at the concentration of 1 μM for 7 days and the observation period of RA induction was based on previously published studies,^37^, ^38^, ^39^ most cells produced synapse‐like protrusion (Figure 1A,B). To further testify the result of differentiation, immunofluorescence staining of known neural stem cell marker nestin and neuron‐specific marker Tuj‐1 were employed. Immunofluorescence staining images revealed that the nearly all C17.2 NSCs without RA treatment exhibited strong positive signal of nestin, whereas the signal of Tuj‐1 could not be detected (Figure 1C,E). After treated with RA for 7 days, around 40% of C17.2 NSCs displayed neuronal‐like morphological change, accompanied by the bright signal of Tuj‐1, and the decrease of nestin (Figure 1D,G–H). Western blotting was applied for further confirm, the results were consistent with those of immunofluorescence staining (Figure 5C,D,F), and the western blotting results also showed that the GFAP expression increased in the RA treatment group (Figure 5C,E). All the results indicated that RA could induce differentiation of C17.2 NSCs into neuron.

### 3.2 LC–MS/MS‐based phosphopeptide and phosphoprotein profiling of RA‐treated C17.2 NSCs

**FIGURE 2:**
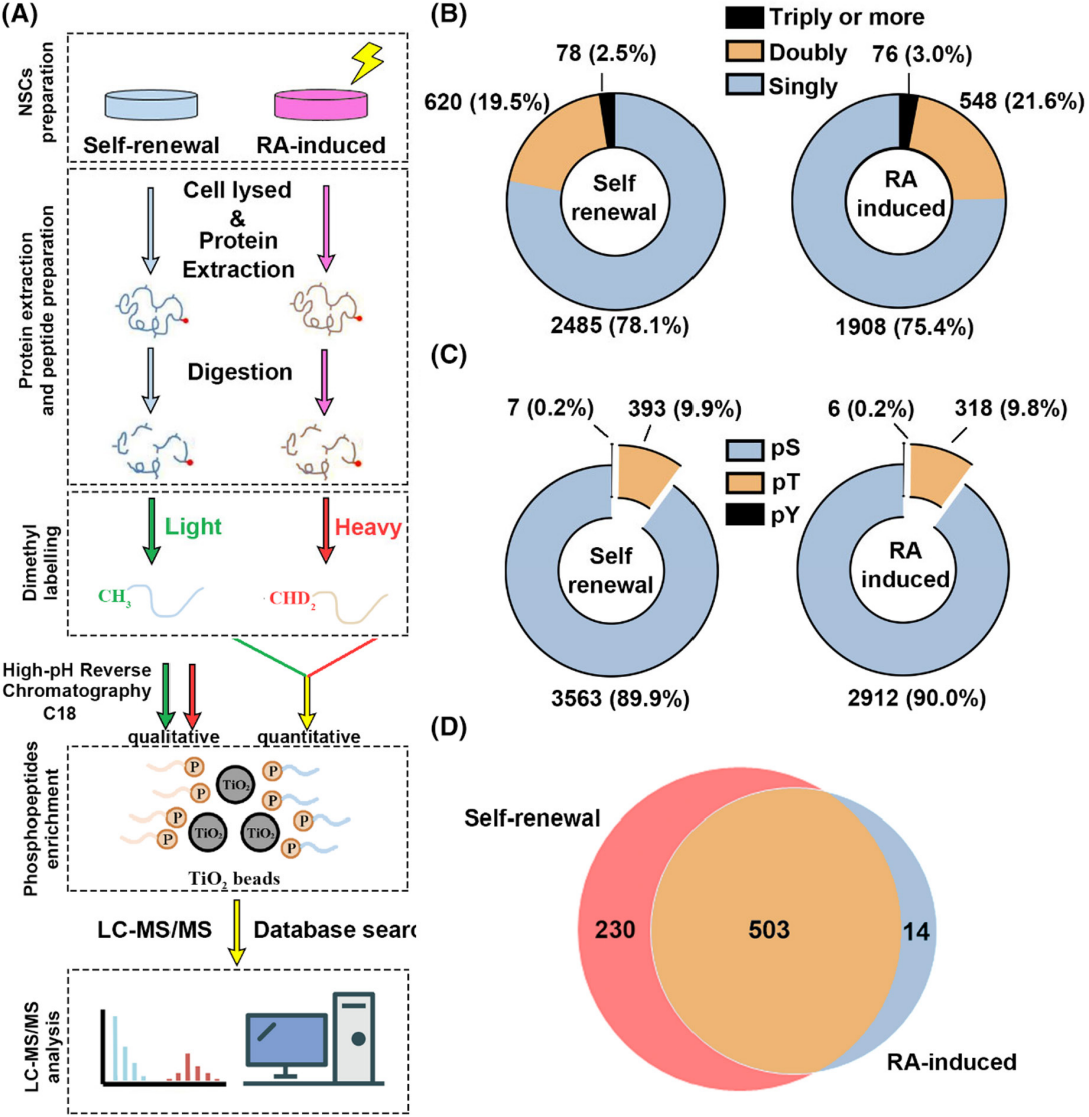
Qualitative phosphoproteome analysis of self‐renewal and RA‐induced group. (A) The proteomic workflow for qualitative and quantitative proteomics: Proteins were extracted from C17.2 NSCs with or without RA treatment. Extracted proteins were digested by trypsin. The digested peptides underwent labelling with isotopomeric dimethyl. Fractions were then separated through C18 chromatography, and phosphopeptides were enriched using TiO_2_ beads before being analysed by reversed‐phase LC–MS/MS (B) Counts of identified phosphopeptides with single, double and triple or more phosphorylation modifications in self‐renewal (left) and RA‐induced (right) group. (C) Counts of identified phosphosites in serine (pS), threonine (pT) and tyrosine (pY) in self‐renewal (left) and RA‐induced (right) group. (D) Venn diagram showing the number of identified unique phosphoproteins in Self‐renewal and RA‐induced group.

To analyse whether the phosphoproteome was affected in C17.2 NSCs with RA treatment, large‐scale qualitative and quantitative phosphoproteome using metal oxide chromatography enrichment method coupled with LC–MS/MS approaches were performed in this study (Figure 2A). The cells were treated with 1 μM RA for 7 days to induce differentiation. C17.2 NSCs were analysed under normal conditions or RA treatment conditions, resulting in two different groups samples named self‐renewal and RA‐induced groups, separately. Total cellular proteins were extracted and digested separately from the two groups, followed by desalting to remove salt ions that could interfere with labelling and mass spectrometry detection. For quantitative phosphoproteome analysis, three peptide samples from each group were chemically labelled with light (L) and heavy (H) dimethyl chemicals, respectively, and then mixed. The resulted samples were fractionated by C18 reversed‐phase high‐performance liquid chromatography. Consecutively, collected fractions were coincubation with metal oxide affinity beads (TiO_2_) to enrich phosphopeptides. Finally, samples were submitted to LC–MS/MS for phosphopeptides identification.

By a standard 1% FDR cut off, qualitative phosphoproteome revealed 2644 unique phosphopeptides in self‐renewal group and 2211 unique phosphopeptides in RA‐induced group. Further analysis of phosphorylation sites, 3963 and 3236 phosphorylation sites were identified in self‐renewal group and RA‐induced group respectively (Table S1). For self‐renewal group, 2485 mono‐phosphorylated peptides, 620 double phosphorylated peptides and 78 more than triple‐phosphorylated peptides were detected (Figure 2B). The identified phosphorylated sides in self‐renewal group contains 89.9% phosphoserine sites, 9.9% phosphothreonine sites and 0.2% phosphotyrosine sites (Figure 2C). For RA‐induced differentiated group, 1908 mono‐phosphorylated peptides, 548 double phosphorylated peptides and 76 triple‐phosphorylated peptides were obtained (Figure 2B). It indicated 90.0% phosphoserine sites, 9.8% phosphothreonine sites and 0.2% phosphotyrosine sites in RA‐induced group (Figure 2C).

In total, 733 unique phosphoproteins were identified in self‐renewal cells while 517 were retrieved from the RA‐induced differentiated cells. Among them, 503 proteins were present in both groups, 230 phosphoproteins only detected in the self‐renewal group and 14 phosphoproteins were only identified in RA‐induced group (Figure 2D). Differentially expressed phosphoproteins that only identified in self‐renewal or RA‐induced differentiated group are listed in Table S2. According to Gene Ontology analysis, two groups showed similar distribution in molecular function (Figure S1). Both groups showed 40% phosphoproteins were involved in RNA transcriptional and proliferation. It indicated that self‐differentiation and RA‐induced differentiation were both mainly regulated by transcription level. Some kinases identified exclusively in self‐renewal group have been reported to be involved in cell cycle and proliferation, including p21‐activated kinases (PAKs), cell division cycle protein 23 (Cdk23) and CDC like kinase 3. For RA‐induced differentiation, phosphorylated Tau, a neurite marker of neurofibrillary tangles and plaque was only presented in differentiated group. Furthermore, ZC3H14, a highly expressed protein in rodent hippocampal neurons and functioned in the regulation RNA metabolism in nervous system cells, was phosphorylated and only identified in RA‐induced group. In addition, p53‐induced protein 11, a protein up‐regulated in apoptosis or cell growth inhibition was also presented in this group. Indeed, RA could induce neural differentiation efficiently; even this process was accompanied with serious neuron apoptosis.

### 3.3 Conserved phosphorylation motif analysis of the phosphorylation sites

The sequence consensus of phosphopeptide motifs reflects the kinase‐specific regulation of substrates and the identification of the corresponding kinases. To find the possible kinases involved in the RA‐transduced downstream phospho‐signals, the flanking sequences of high‐confident phosphorylation residues were submitted to Motif‐X,^33^ and the frequency of particular amino acids in the proximity of phosphorylation sites was analysed. In total, 2644 and 2211 unique phosphopeptites from the self‐renewal and RA‐induced group were subjected to the motif X analysis. A motif width of 13, an occurrences threshold of 20 and a significance of 10^‐6^ have been chosen in this operation.

**FIGURE 3:**
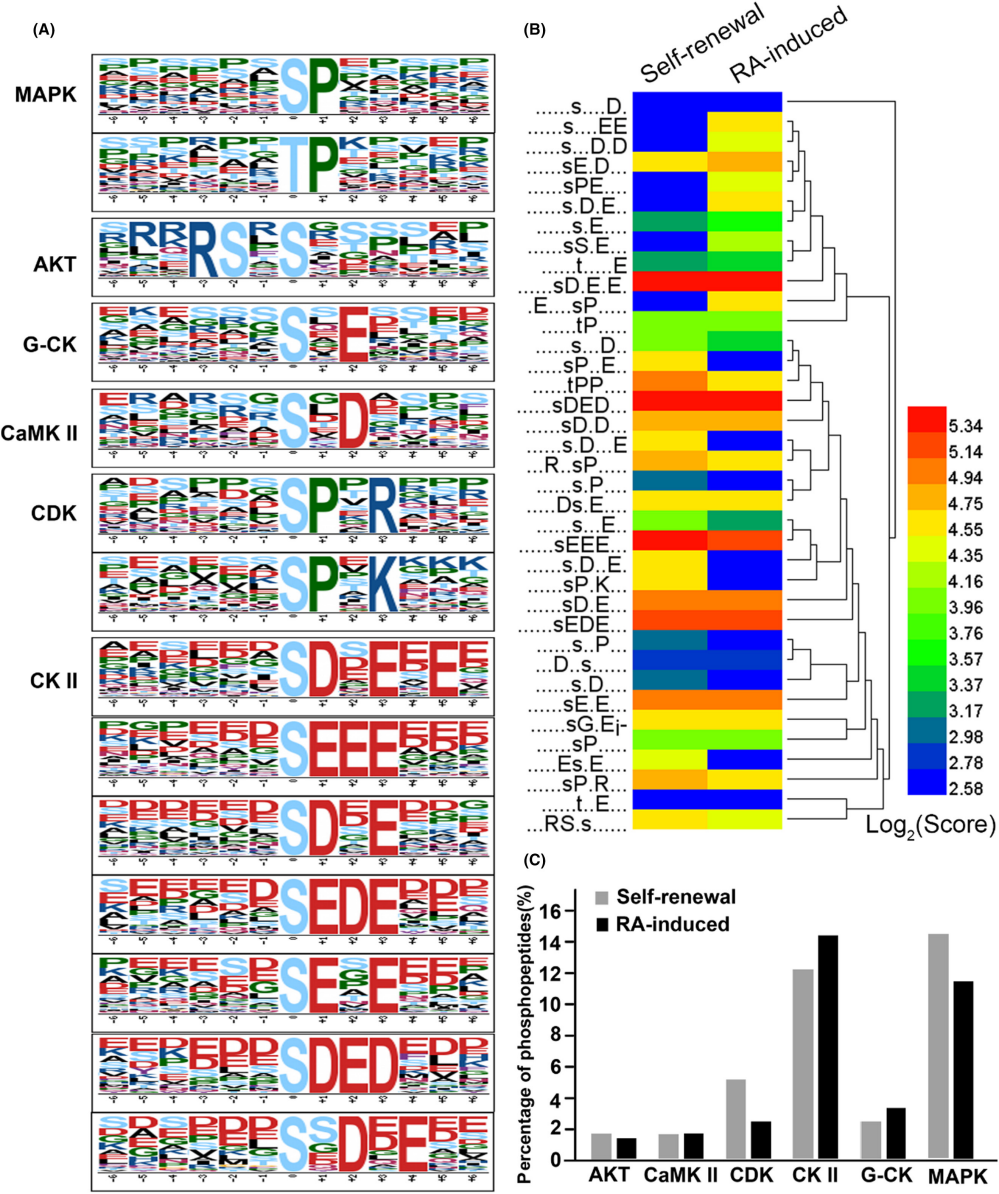
Motif‐X analysis of phosphorylation motifs. (A) The highly conserved phosphosites motifs in both self‐renewal and RA‐induced groups. These motifs are corresponding to casein kinase II (CKII), CaMK II, Golgi casein kinase (G‐CK), cyclin‐dependent kinase (CDK) and mitogen‐activated protein kinase (MAPK). (B) Comparison of motif enrichment score in self‐renewal and RA‐induced group. (C) Comparison of the corresponding kinases of motifs enriched in self‐renewal and RA‐induced group.

As the result, more than 13 over‐represented motifs were enriched from the phosphosites in both groups (Figure 3A). Through literature and database survey, some motifs corresponding kinases are well‐known: pS[D/E]X[D/E], pSEEE, pSEDE and pSDED for casein kinase II; pSXD for CaMK II; pSXE for golgi casein kinase (G‐CK); and pSPX[R/K] for cyclin‐dependent kinase (CDK). p[S/T]P for mitogen‐activated protein kinase (MAPK) families. Besides, pS/T–P is a known target of the proline‐directed kinase was over‐represented in both groups. It is necessary to point out that R‐X‐X‐Ps, the only basic motif is a target of CaMK II, which may have a significant role in the NSCs. It has been reported that CaMK II is involved in synaptic transmission, plasticity and NSCs differentiation.^40^, ^41^

Sequentially, we compared motifs in two groups with score profiles to reveal the changes in RA‐induced differentiation (Figure 3B,C). Motif recognized by MAPK and casein kinase II are most enriched in both groups. The phosphorylation levels of proteins containing G‐CK recognizing motifs pSXE tend to be up‐regulated after RA treatment, while MAPK families recognizing motifs p[S/T]P and CDK recognizing motifs pSPX[R/K] were down‐regulated in RA‐induced group. The results showed that golgi casein kinase, cyclin‐dependent kinase and MAPK signalling pathway are related to RA‐induced differentiation. However, the CK2‐related phosphosites with motif pSDED, pSDX[D/E] and pSEXE showed no significant different between self‐renewal and RA‐induced group, indicating that CK2 played a crucial role in growth of stem cell but unrelated with RA.

### 3.4 GO and pathway analysis of differential phosphoproteome

According to the result of above qualitative proteomics, protein phosphorylation plays critical roles in the process of NSCs RA‐induced differentiation. To further investigate the changes of phosphorylated levels after RA‐treatment, quantitative phosphoproteome based on dimethyl labelling were employed to compare the phosphoproteome change between the self‐renewal cells and the neural differentiating cells induced by RA. Three biological replicates were collected for each sample. The self‐renewal and RA‐induced samples could be clustered into two categories in the PCA analysis (Figure S2A). In addition, the correlation between each replicate of self‐renewal (light labelled) reached a Pearson correlation of 0.907 and 0.906. The correlation between each replicate of RA‐induced (heavy labelled) reached a Pearson correlation of 0.665 and 0.681 (Figure S2B). Overall, these findings indicate the reliability of the quantitative proteomics data set obtained from the three biological replicates.

**FIGURE 4:**
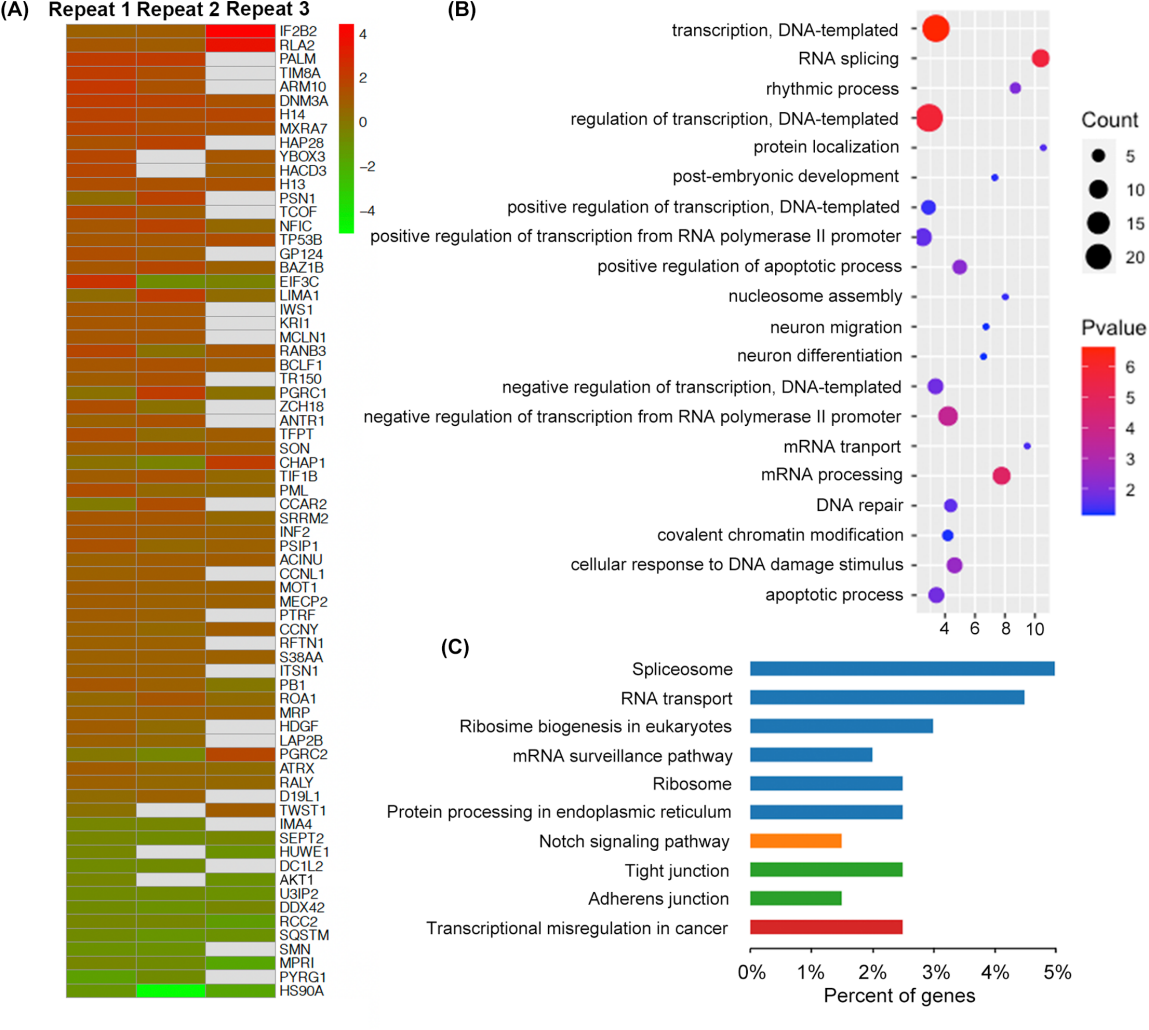
Analysis of differentially expressed phosphorylated proteins and related to RA. (A) Heat map showing the log2 intensity ratios for the differentially expressed phosphoproteins that were detected in least two replications. Increased or decreased abundance of proteins under RA treatment is represented with the red to blue colour scale. (B) GO analysis of the differentially expressed phosphorylated proteins. (C) Pathway enrichment of the differentially expressed phosphorylated proteins.

A total of 2340 unique phosphopeptides were successfully quantified. Their average ratios and standard deviations were calculated from three replicates. Among them, 177 unique phosphoproteins were identified were successfully quantified in least two replicates (Table S3). The L/H ratio of phosphoproteins expressions in the two groups were visualized using heat maps, to intuitively display the phosphorylation level changes in RA induced (Figure 4A). Seventy phosphoproteins showed differentially expression including 57 up‐regulated and 13 down‐regulated phosphoproteins. To gain the functional insights into RA and its associated pathways, GO enrichment and KEGG pathway enrichment analysis were performed on these differential phosphoproteins (Figure 4B,C). The GO analysis revealed that the RA‐induced regulated phosphoproteins are primarily involved in transcription and RNA splicing as part of the biological process. In terms of cell components, most of phosphoproteins are primarily localized in the nucleus. The molecular function of differential phosphoproteins is related to nucleic acid binding and protein binding. It is indicated that the identified differential phosphoproteins are mainly related to regulation of nuclear. It was likely to contribute to signal delivers to nucleus for transcription after RA treatment. Pathway enrichment analysis also showed signalling pathways of RNA transport and spliceosome were significantly enriched. It also further showed that the regulation of RA‐induced differentiation mainly occurred at the transcription level.

### 3.5 Protein–protein interaction network analysis of RA‐related phosphorylated proteins

**FIGURE 5:**
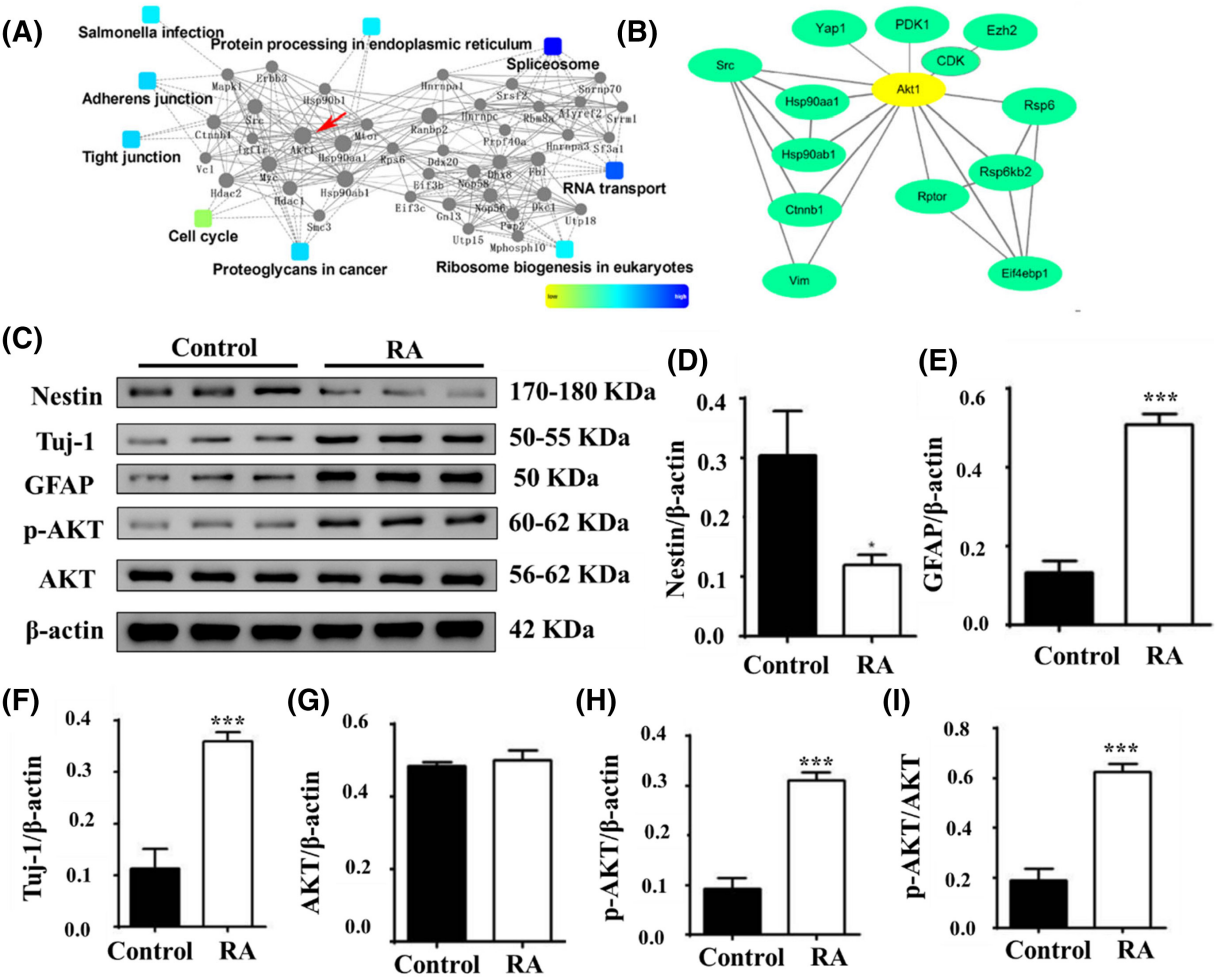
Protein–protein interaction network analysis of RA‐related phosphorylated proteins. (A) Protein–protein interaction network among differentially expressed phosphorylated proteins. The sizes of nodes represented the number of interactions. Clustering analysis was done according to GO annotation. (B) Protein–protein interaction network with core protein AKT1 according to literature and database survey. (C–I) Western blotting results of Nestin, Tuj‐1, GFAP, AKT and p‐AKT expression in C17.2 NSCs with/without RA treatment. *n* = 3 per group, **p < 0.01, ***p < 0.001, compared with the control group.

To further analyse the downstream pathways of RA signal, protein–protein interaction network (PIN) analysis was performed on differentially expressed phosphoproteins (Figure 5A). GO analysis indicated proteins' involvement in some specific functions, such as adherens junction, tight junction, RNA transport, protein processing in the endoplasmic reticulum, proteoglycans in cancer, Salmonella infection, and cell cycle, especially, IMP2 and Twist1 (Figure 5A; Figure S3). IMP2 is implicated in cell proliferation, migration and apoptosis,^42^ while Twist1 regulates apoptosis, cell cycle and cell differentiation.^43^ The findings of this study provide new insights into the functions of these proteins in the differentiation of NSCs. From the network, we found that many phosphoproteins identified in this study were known or strongly associated with NSCs proliferation and differentiation, including AKT, Src, PAKs and β‐catenin. In addition, AKT1 acts as a core component with 15 interactions in PIN analysis. Studies showed AKT1 plays important roles in neuron differentiation. To further investigate the role of AKT1 in RA‐induced neuronal differentiation, interaction of AKT1 were analysed by the literature and STRING database survey (Figure 5B). The result showed AKT1 interact with Src to co‐regulate Hsp90, β‐catenin and vim activity, and further regulate cell proliferation and differentiation. In addition, AKT1 can be phosphorylated by PDK1, and it is also the kinase of YAP. AKT also can phosphorylate CDK to regulate the activity of Ezh2, which contributes to inhibit cell proliferation and promote differentiation. Recent studies have shown that AKT1 plays a very important role in cell survival and apoptosis. Some growth and survival factors (such as insulin‐like growth factor) can activate the AKT signalling pathway. It implied that AKT1 played a significant role in regulation of RA‐induced cell differentiation. The expression of AKT after treated with RA for 7 days was detected by western blotting. The results demonstrated a significant increase in the expression of p‐AKT, but no change in the expression of total AKT, compared to the untreated groups (Figure 5C,H–I). Collectively, these findings highlight the crucial role of AKT signalling in RA‐induced differentiation of C17.2 NSCs.

## 4 DISCUSSION

RA signalling has been identified as a key driver in stem cell differentiation. RA stimulated the signalling by binding to nuclear receptor which functions as ligand‐dependent transcription factors, then regulated the expression of RA responsive genes for differentiation processes. In this study, comparative qualitative and quantitative phosphoproteomics were performed to reveal the potential proteins of RA signalling. Our phosphoproteomic analysis identified known and novel proteins and phosphorylation sites potentially participating in self‐renewal or differentiation of C17.2 NSCs. As a result of qualitative phosphoproteomics, we identified 733 and 517 unique phosphoproteins, respectively, from the self‐renewal and RA‐induced differentiated cells. Further investigation of changes in phosphorylation level was performed on quantitative phosphoproteomics. A total of 2340 unique phosphopeptides were successfully quantified, derived from 177 phosphoproteins. Among them, 70 phosphoproteins showed significantly differentially expression, including 57 up‐regulated and 13 down‐regulated phosphoproteins.

Interestingly, only 14 phosphoproteins were exclusively identified in the RA‐induced group, while most of the phosphoproteins were detected in both groups. The result suggested that differentiation by RA induction has the same biological process as normal differentiation. Furthermore, the RA‐induced group exhibited a similar distribution in molecular function compared to the self‐renewal group, as revealed by Gene Ontology (GO) analysis, which further supports the similarity between RA‐induced and normal differentiation. Notably, GO analysis indicated that the majority of the identified phosphoproteins are involved in transcription, indicating that the differentiation process of stem cells is primarily regulated at the transcriptional level. Motif analysis on the identified phosphopeptide sequences revealed that the most enriched motif in two groups can be recognized by the same kinase (CKII and MAPK), which also can support that RA‐induced differentiation is similar with normal differentiation. CDK recognizing motifs showed significant down‐regulation after RA treatment. CDKs play important roles in the control of cell division in response to several extra‐ and intracellular signal. CDK can regulate the cell cycle and get involved in the regulation of transcription.^44^ Therefore, we speculated that RA may affect the differentiation of NSCs by regulating the cell cycle. According to the analysis of quantitative and quantitative phosphoproteomics, significant changed phosphoproteins regulated by RA induction were screened. According to GO and PIN analysis, some phosphoproteins were indeed related to the transcription of cell cycle proteins, such as insulin‐like growth factor 2 mRNA‐binding protein 2 (IMP2) and Twist1. Evidence showed that IMP2 could mediate translation of proliferation genes such as N‐Ras and c‐Myc and over‐expression of IMP2 increased the neurogenic potential and suppressed astrocytic differentiation.^45^, ^46^ Additionally, Ser162 and Ser164 of IMP2, which were also identified in this study, were simultaneously phosphorylated by mTOR to promote the translation of IGF2.^47^ In our results, the phosphorylation level of Ser162 and Ser164 on IMP2 were both increased in RA‐induced group. One possible reason was that IMP2 was inactivated with phosphorylation and promote IGF2 expression and C17.2 NSCs differentiation. Twist1 bound to the promoter regions of cyclin E1, E2F1 and c‐Myc suggests a direct transcriptional regulation.^48^, ^49^ Twist1 over‐expression could promote odontoblast terminal differentiation^50^ and regulate cell fate determination between ectodermal and mesodermal lineages.^51^ Phosphoregulation of Twist1 controlled cell fate, but whether Twist1 regulated neural stem cell differentiation had no report. And the activity of Twist1 was regulated by MAPK‐mediated phosphorylation of Ser68.^52^ The data set showed that p‐S68 of Twist1 was increased in RA‐induced group. Possibly, phosphorylation of Twist1 (p‐S68) may promote neuron differentiation.

Evidence suggests that the Wnt/β‐catenin pathway affects neuronal fate determination and induces key neuronal markers. By inhibiting this pathway, NSC differentiation is prevented, resulting in the non‐division of the cells.^53^ According to our pathway enrichment and PIN analysis, AKT act as a core protein in the network. AKT can be phosphorylated by PDK1 in Thr 308 and activate the kinase activity of CDK.^54^, ^55^ Activated CDK inhibit EzH2, resulting in the inhibition of proliferation and promotion of differentiation.^56^, ^57^, ^58^ In addition, AKT had correlation with several Wnt signalling‐associated proteins which were both detected in this study, including β‐catenin, PAK1, PAK2, Merlin, EzH2 and Src. Wnt/β‐catenin signalling pathway played multiple roles in a variety of cellular processes including development, cell proliferation, cell fate and motility. As a key effector of Wnt pathway, the β‐catenin played a major role in the regulation of cell adhesion and gene transcription. The post‐translational modification, especially phosphorylation, is a significant regulation mechanism of β‐catenin. The stability, function and localization of β‐catenin all had correlation with the phosphorylation state. The phosphorylation peptides containing S675, S552 and S191 in β‐catenin were identified in this study and might be involved in the function of β‐catenin.^59^ It has been reported that merlin, PAKs, AKT and Src could regulate NSCs proliferation and differentiation by regulating stability, function and localization of β‐catenin via phosphorylation.^60^, ^61^, ^62^, ^63^ Research showed that PAK2 regulated the β‐catenin cellular localization on plasma membrane by phosphorylating the S518 site of the substrate merlin.^64^, ^65^ Phosphorylated merlin lost the functions of maintaining β‐catenin at the plasma membrane, resulting in the release of β‐catenin and translocation into cytoplasm.^62^ AKT phosphorylated β‐catenin at Ser552 in cytoplasm, which in turn, promoted its nuclear localization and transcriptional activity. PAK1, as the substrate of CDK11, could phosphorylate cortactin and β‐catenin.^66^, ^67^, ^68^ Src can help β‐catenin into the nucleus. β‐catenin reported phosphorylated Y654 by Src and activated expression of c‐myc and cyclin D1 which functions as regulator of differentiation progress.^69^, ^70^ In this study, the phosphorylation sites of Wnt/β‐catenin signalling‐associated proteins were detected. Our results indicated that RA signalling may promote differentiation progress through regulation of β‐catenin.

In addition, AKT also had correlation with Hippo signalling pathway. AKT is a kinase of YAP protein, which is the downstream effector molecule in the Hippo signalling pathway.^71^ Studies on the Hippo signalling pathway also show involvement in NSC differentiation. Activation of the Hippo pathway promotes neurogenesis and enhances neuron generation from NSCs.^72^ Inhibition of the Hippo pathway, on the other hand, leads to decreased neurogenesis and NSC maintenance in an undifferentiated state.^73^ Several Hippo signalling‐associated proteins such as F‐actin, Merlin and Mst 1/2 were detected in our study. 14‐3‐3 protein can be phosphorylated by α‐catenin and helps interaction with YAP protein. YAP bound to 14‐3‐3 protein inhibit transcriptional regulation activity of YAP.^74^ With RA induction, merlin and F‐actin can phosphorylate Mst1/2, which is the core enzyme in the Hippo signalling pathway.^75^, ^76^ Phosphorylated Mst1/2 inhibited phosphorylation of YAP and the transcriptional regulation activity of YAP was activated.^77^ Our results suggested that RA may promote differentiation progress by regulation of YAP signalling pathway. It is worth mentioning that RA‐induced neural differentiation was accompanied by neural apoptosis. Phosphorylation of apoptosis regulator 2 which is phosphorylated by the apical kinases ATM/ATR may enhance SIRT1 inhibition, p53 acetylation and p53‐dependent apoptosis.^78^ Six phosphorylated sites from apoptosis regulator 2 were identified in this study. Among them, Thr606 and Ser677 were up‐regulated while Ser680 was down‐regulated in RA‐induced group, which may contribute to neural apoptosis. However, this study had inevitable limitations. A limitation was the small range of samples tested for analysis, which may have skewed the results. Furthermore, this study was not validated by further expanding the western blotting validations and in vivo experiments, which may have impacted the results. Therefore, further experiments should be conducted to confirm these findings. Our future study will focus on the roles of these molecules during neuronal differentiation. This research could help us better understand the role of RA in neuronal differentiation.

**FIGURE 6:**
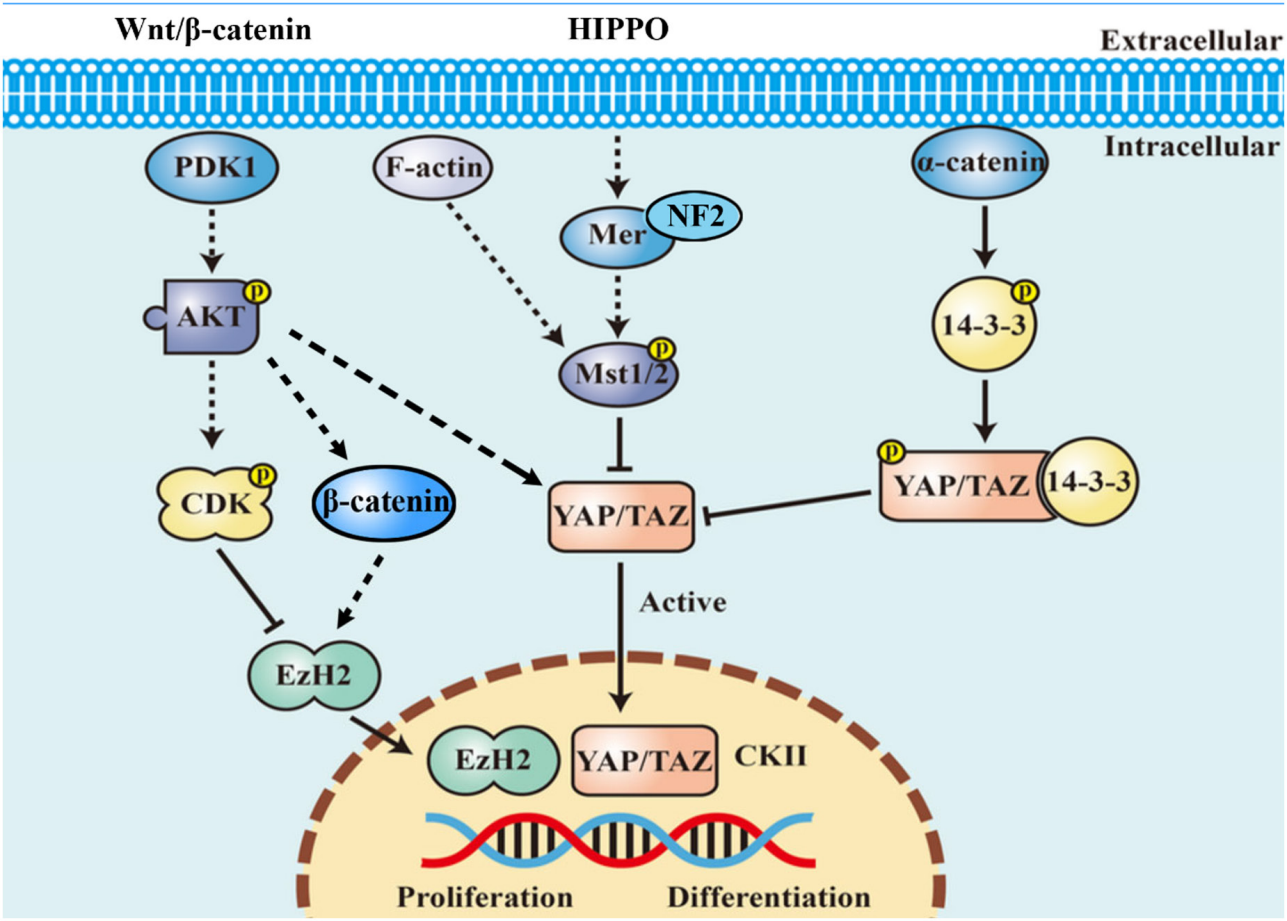
Model of RA‐induced neuronal differentiation. This model is based on the information provided in this study and references cited in the Discussion. The solid line represented relationships that were obtained from literature. Dotted line represented hypothesized relationships from this study.

In this article, we performed qualitative and quantitative phosphoproteomics to study RA‐induced neuronal differentiation. According to the results of the phosphoproteomics, we proposed a model in which AKT acted as a core regulator in RA‐induced neuronal differentiation (Figure 6). RA treatment increased AKT phosphorylation and regulated neuronal differentiation by Wnt/β‐catenin and Hippo signalling pathway.

## AUTHOR CONTRIBUTIONS

**Cheng Zhang:** Formal analysis (equal); validation (equal); writing – original draft (equal). **Lite Ge:** Formal analysis (equal); validation (equal); writing – original draft (equal). **Huali Xie:** Validation (equal). **Xiaoqian Liu:** Investigation (equal). **Chengfeng Xun:** Validation (equal). **Yan Chen:** Validation (equal). **Haiyan Chen:** Writing – review and editing (equal). **Ming Lu:** Conceptualization (equal). **Ping Chen:** Conceptualization (equal).

## FUNDING INFORMATION

This work was supported by Changsha Natural Science Foundation Project (Grant No.76834), National Natural Science Foundation of China (No. 31670838, No.32000151 and No.81974213) and China Postdoctoral Science Foundation (2020 M672675).

## CONFLICT OF INTEREST STATEMENT

No conflict of interest exits in the submission of this article.

## Supporting information


Figure S1.
Figure S2.
Figure S3.

Table S1.

Table S2.

Table S3.

## Data Availability

All data, models and code generated or used during this study appear in the submitted article.

## References

[1] Temple S . The development of neural stem cells. Nature. 2001;414(6859):112‐117. 11689956 10.1038/35102174

[2] Gage FH . Mammalian neural stem cells. Science. 2000;287(5457):1433‐1438. 10688783 10.1126/science.287.5457.1433

[3] Lutfi Ismaeel G , Makki AlHassani OJ , S. Alazragi R , et al. Genetically engineered neural stem cells (NSCs) therapy for neurological diseases; state‐of‐the‐art. Biotechnol Prog. 2023;39:e3363. 37221947 10.1002/btpr.3363

[4] Ransom J , Morgan PJ , McCaffery PJ , Stoney PN . The rhythm of retinoids in the brain. J Neurochem. 2014;129(3):366‐376. 24266881 10.1111/jnc.12620 PMC4283048

[5] Szutowicz A , Bielarczyk H , Jankowska‐Kulawy A , Ronowska A , Pawelczyk T . Retinoic acid as a therapeutic option in Alzheimer's disease: a focus on cholinergic restoration. Expert Rev Neurother. 2015;15(3):239‐249. 25683350 10.1586/14737175.2015.1008456

[6] Gutierrez‐Mazariegos J , Schubert M , Laudet V . Evolution of retinoic acid receptors and retinoic acid signaling. Subcell Biochem. 2014;70:55‐73. 24962881 10.1007/978-94-017-9050-5_4

[7] Mohr H , Ballke S , Bechmann N , et al. Mutation of the cell cycle regulator p27kip1 drives pseudohypoxic pheochromocytoma development. Cancers (Basel). 2021;13(1):126. 33401758 10.3390/cancers13010126 PMC7794757

[8] Matsuo T , Thiele CJ . p27Kip1: a key mediator of retinoic acid induced growth arrest in the SMS‐KCNR human neuroblastoma cell line. Oncogene. 1998;16(25):3337‐3343. 9681834 10.1038/sj.onc.1201830

[9] Dimberg A , Oberg F . Retinoic acid‐induced cell cycle arrest of human myeloid cell lines. Leuk Lymphoma. 2003;44(10):1641‐1650. 14692514 10.1080/1042819031000083316

[10] Hedblom A , Laursen KB , Miftakhova R , et al. CDK1 interacts with RARgamma and plays an important role in treatment response of acute myeloid leukemia. Cell Cycle. 2013;12(8):1251‐1266. 23518499 10.4161/cc.24313 PMC3674090

[11] Kuang Y , Kang J , Li H , et al. Multiple functions of p21 in cancer radiotherapy. J Cancer Res Clin Oncol. 2021;147(4):987‐1006. 33547489 10.1007/s00432-021-03529-2 PMC11802169

[12] Shibata M , Pattabiraman K , Lorente‐Galdos B , et al. Regulation of prefrontal patterning and connectivity by retinoic acid. Nature. 2021;598(7881):483‐488. 34599305 10.1038/s41586-021-03953-x PMC9018119

[13] Marshall H , Nonchev S , Sham MH , Muchamore I , Lumsden A , Krumlauf R . Retinoic acid alters hindbrain Hox code and induces transformation of rhombomeres 2/3 into a 4/5 identity. Nature. 1992;360(6406):737‐741. 1361214 10.1038/360737a0

[14] Maden M . Retinoic acid in the development, regeneration and maintenance of the nervous system. Nat Rev Neurosci. 2007;8(10):755‐765. 17882253 10.1038/nrn2212

[15] Kaplan DR , Matsumoto K , Lucarelli E , Thielet CJ . Induction of TrkB by retinoic acid mediates biologic responsiveness to BDNF and differentiation of human neuroblastoma cells. Neuron. 1993;11(2):321‐331. 8394722 10.1016/0896-6273(93)90187-v

[16] Janesick A , Wu SC , Blumberg B . Retinoic acid signaling and neuronal differentiation. Cell Mol Life Sci. 2015;72(8):1559‐1576. 25558812 10.1007/s00018-014-1815-9 PMC11113123

[17] Gong M , Bi Y , Jiang W , et al. Retinoic acid receptor beta mediates all‐trans retinoic acid facilitation of mesenchymal stem cells neuronal differentiation. Int J Biochem Cell Biol. 2013;45(4):866‐875. 23318218 10.1016/j.biocel.2013.01.002

[18] De La Rosa‐Reyes V , Duprey‐Diaz MV , Blagburn JM , Blanco RE . Retinoic acid treatment recruits macrophages and increases axonal regeneration after optic nerve injury in the frog Rana pipiens. PLoS One. 2021;16(11):e0255196. 34739478 10.1371/journal.pone.0255196 PMC8570512

[19] Sodhi RK , Singh N . Retinoids as potential targets for Alzheimer's disease. Pharmacol Biochem Behav. 2014;120:117‐123. 24582848 10.1016/j.pbb.2014.02.016

[20] Shudo K , Fukasawa H , Nakagomi M , Yamagata N . Towards retinoid therapy for Alzheimer's disease. Curr Alzheimer Res. 2009;6(3):302‐311. 19519313 10.2174/156720509788486581 PMC2765081

[21] Clark JN , Whiting A , McCaffery P . Retinoic acid receptor‐targeted drugs in neurodegenerative disease. Expert Opin Drug Metab Toxicol. 2020;16(11):1097‐1108. 32799572 10.1080/17425255.2020.1811232

[22] Cao Q‐L , Zhang YP , Howard RM , Walters WM , Tsoulfas P , Whittemore SR . Pluripotent stem cells engrafted into the normal or lesioned adult rat spinal cord are restricted to a glial lineage. Exp Neurol. 2001;167(1):48‐58. 11161592 10.1006/exnr.2000.7536

[23] Fairless R , Barnett SC . Olfactory ensheathing cells: their role in central nervous system repair. Int J Biochem Cell Biol. 2005;37(4):693‐699. 15694828 10.1016/j.biocel.2004.10.010

[24] Ramón‐Cueto A , Valverde F . Olfactory bulb ensheathing glia: a unique cell type with axonal growth‐promoting properties. Glia. 2004;14(3):163‐173. 10.1002/glia.440140302 7591028

[25] Barnett SC . Identification of a human olfactory ensheathing cell that can effect transplant‐mediated remyelination of demyelinated CNS axons. Brain. 2000;123(8):1581‐1588. 10908188 10.1093/brain/123.8.1581

[26] Ramer LM , Au E , Richter MW , Liu J , Tetzlaff W , Roskams AJ . Peripheral olfactory ensheathing cells reduce scar and cavity formation and promote regeneration after spinal cord injury. J Comp Neurol. 2004;473(1):1‐15. 15067714 10.1002/cne.20049

[27] Deumens R , Koopmans GC , Honig WMM , et al. Olfactory ensheathing cells, olfactory nerve fibroblasts and biomatrices to promote long‐distance axon regrowth and functional recovery in the dorsally hemisected adult rat spinal cord. Exp Neurol. 2006;200(1):89‐103. 16527274 10.1016/j.expneurol.2006.01.030

[28] Snyder EY , Deitcher DL , Walsh C , Arnold‐Aldea S , Hartwieg EA , Cepko CL . Multipotent neural cell lines can engraft and participate in development of mouse cerebellum. Cell. 1992;68(1):33‐51. 1732063 10.1016/0092-8674(92)90204-p

[29] Wang S , Li Z , Shen H , et al. Quantitative Phosphoproteomic study reveals that protein kinase a regulates neural stem cell differentiation through phosphorylation of catenin Beta‐1 and glycogen synthase kinase 3β. Stem Cells. 2016;34(8):2090‐2101. 27097102 10.1002/stem.2387

[30] Wiśniewski JR , Zougman A , Nagaraj N , Mann M . Universal sample preparation method for proteome analysis. Nat Methods. 2009;6(5):359‐362. 19377485 10.1038/nmeth.1322

[31] Boersema PJ , Raijmakers R , Lemeer S , Mohammed S , Heck AJ . Multiplex peptide stable isotope dimethyl labeling for quantitative proteomics. Nat Protoc. 2009;4(4):484‐494. 19300442 10.1038/nprot.2009.21

[32] Nguyen EV , Imanishi SY , Haapaniemi P , et al. Quantitative site‐specific Phosphoproteomics of Trichoderma reesei signaling pathways upon induction of hydrolytic enzyme production. J Proteome Res. 2016;15(2):457‐467. 26689635 10.1021/acs.jproteome.5b00796

[33] Schwartz D , Chou MF , Church GM . Predicting protein post‐translational modifications using meta‐analysis of proteome scale data sets. Mol Cell Proteomics. 2009;8(2):365‐379. 18974045 10.1074/mcp.M800332-MCP200 PMC2634583

[34] Huang DW , Sherman BT , Lempicki RA . Systematic and integrative analysis of large gene lists using DAVID bioinformatics resources. Nat Protoc. 2008;4(1):44‐57. 10.1038/nprot.2008.211 19131956

[35] Kanehisa M , Goto S , Sato Y , Furumichi M , Tanabe M . KEGG for integration and interpretation of large‐scale molecular data sets. Nucleic Acids Res. 2011;40(D1):D109‐D114. 22080510 10.1093/nar/gkr988 PMC3245020

[36] Szklarczyk D , Franceschini A , Wyder S , et al. STRING v10: protein–protein interaction networks, integrated over the tree of life. Nucleic Acids Res. 2015;43(D1):D447‐D452. 25352553 10.1093/nar/gku1003 PMC4383874

[37] Zhao M , Chen S , Yang M‐L , Li S‐Y , Jiang W , Xiao N . Vitamin a regulates neural stem cell proliferation in rats after hypoxic‐ischemic brain damage via RARɑ‐mediated modulation of the β‐catenin pathway. Neurosci Lett. 2020;727:134922. 32205185 10.1016/j.neulet.2020.134922

[38] Zhao H , Zuo X , Ren L , et al. Combined use of bFGF/EGF and all‐trans‐retinoic acid cooperatively promotes neuronal differentiation and neurite outgrowth in neural stem cells. Neurosci Lett. 2019;690:61‐68. 30300683 10.1016/j.neulet.2018.10.002

[39] Chu T , Zhou H , Wang T , et al. In vitro characteristics of Valproic acid and all‐trans‐retinoic acid and their combined use in promoting neuronal differentiation while suppressing astrocytic differentiation in neural stem cells. Brain Res. 2015;1596:31‐47. 25463022 10.1016/j.brainres.2014.11.029

[40] Liang C , Du F , Wang J , Cang J , Xue Z . Propofol regulates neural stem cell proliferation and differentiation via Calmodulin‐dependent protein kinase II/AMPK/ATF5 signaling Axis. Anesth Analg. 2019;129(2):608‐617. 30303867 10.1213/ANE.0000000000003844

[41] Nilmani , D'Costa M , Bothe A , et al. CDK regulators—Cell cycle progression or apoptosis—Scenarios in normal cells and cancerous cells. Adv Protein Chem Struct Biol. 2023;135:125‐177. 37061330 10.1016/bs.apcsb.2022.11.008

[42] Degrauwe N , Suvà M‐L , Janiszewska M , Riggi N , Stamenkovic I . IMPs: an RNA‐binding protein family that provides a link between stem cell maintenance in normal development and cancer. Genes Dev. 2016;30(22):2459‐2474. 27940961 10.1101/gad.287540.116 PMC5159662

[43] Rose CSP , Malcolm S . A TWIST in development. Trends Genet. 1997;13(10):384‐387. 9351337 10.1016/s0168-9525(97)01296-1

[44] Malumbres M . Cyclin‐dependent kinases. Genome Biol. 2014;15(6):122. 25180339 10.1186/gb4184 PMC4097832

[45] Fujii Y , Kishi Y , Gotoh Y . IMP2 regulates differentiation potentials of mouse neocortical neural precursor cells. Genes Cells. 2013;18(2):79‐89. 23331702 10.1111/gtc.12024

[46] Gong C , Li Z , Ramanujan K , et al. A long non‐coding RNA, LncMyoD, regulates skeletal muscle differentiation by blocking IMP2‐mediated mRNA translation. Dev Cell. 2015;34(2):181‐191. 26143994 10.1016/j.devcel.2015.05.009

[47] Dai N , Rapley J , Angel M , Yanik MF , Blower MD , Avruch J . mTOR phosphorylates IMP2 to promote IGF2 mRNA translation by internal ribosomal entry. Genes Dev. 2011;25(11):1159‐1172. 21576258 10.1101/gad.2042311 PMC3110954

[48] Srivastava J , Rho O , Youssef RM , DiGiovanni J . Twist1 regulates keratinocyte proliferation and skin tumor promotion. Mol Carcinog. 2016;55(5):941‐952. 26013710 10.1002/mc.22335 PMC4662634

[49] Maciejewska I , Sakowicz‐Burkiewicz M , Krzeminska M , Pawelczyk T . Overexpression of ID1 reverses the repression of human dental pulp stem cells differentiation induced by TWIST1 silencing. Acta Biochim Pol. 2017;64(4):615‐619. 29159326 10.18388/abp.2017_1522

[50] Li Y , Lu Y , Maciejewska I , Galler KM , Cavender A , D'Souza RN . TWIST1 promotes the odontoblast‐like differentiation of dental stem cells. Adv Dent Res. 2011;23(3):280‐284. 21677079 10.1177/0022034511405387 PMC3144037

[51] Vincentz JW , Firulli BA , Lin A , Spicer DB , Howard MJ , Firulli AB . Twist1 controls a cell‐specification switch governing cell fate decisions within the cardiac neural crest. PLoS Genet. 2013;9(3):e1003405. 23555309 10.1371/journal.pgen.1003405 PMC3605159

[52] Sun T , Fu J , Shen T , et al. The small C‐terminal domain phosphatase 1 inhibits cancer cell migration and invasion by dephosphorylating Ser(P)68‐Twist1 to accelerate Twist1 protein degradation. J Biol Chem. 2016;291(22):11518‐11528. 26975371 10.1074/jbc.M116.721795 PMC4882423

[53] Russell JO , Monga SP . Wnt/β‐catenin signaling in liver development, homeostasis, and pathobiology. Annu Rev Pathol: Mech Dis. 2018;13(1):351‐378. 10.1146/annurev-pathol-020117-044010 PMC5927358 29125798

[54] Zeng T , Zhang CL , Zhao N , et al. Impairment of Akt activity by CYP2E1 mediated oxidative stress is involved in chronic ethanol‐induced fatty liver. Redox Biol. 2018;14:295‐304. 28987868 10.1016/j.redox.2017.09.018 PMC5633250

[55] Xie S , Wei F , Sun YM , et al. EZH2 inhibitors abrogate upregulation of trimethylation of H3K27 by CDK9 inhibitors and potentiate its activity against diffuse large B‐cell lymphoma. Haematologica. 2020;105(4):1021‐1031. 31289198 10.3324/haematol.2019.222935 PMC7109751

[56] Wu SC , Zhang Y . Cyclin‐dependent kinase 1 (CDK1)‐mediated phosphorylation of enhancer of zeste 2 (Ezh2) regulates its stability. J Biol Chem. 2011;286(32):28511‐28519. 21659531 10.1074/jbc.M111.240515 PMC3151093

[57] Wei Y , Chen YH , Li LY , et al. CDK1‐dependent phosphorylation of EZH2 suppresses methylation of H3K27 and promotes osteogenic differentiation of human mesenchymal stem cells. Nat Cell Biol. 2011;13(1):87‐94. 21131960 10.1038/ncb2139 PMC3076036

[58] Yang Y , Dai Y , Yang X , Wu S , Wang Y . DNMT3A mutation‐induced CDK1 overexpression promotes Leukemogenesis by modulating the interaction between EZH2 and DNMT3A. Biomol Ther. 2021;11(6):781. 10.3390/biom11060781 PMC8224654 34067359

[59] Park MH , Kim DJ , You ST , et al. Phosphorylation of β‐catenin at serine 663 regulates its transcriptional activity. Biochem Biophys Res Commun. 2012;419(3):543‐549. 22369945 10.1016/j.bbrc.2012.02.056

[60] Selamat W , Tay PL , Baskaran Y , Manser E . The Cdc42 effector kinase PAK4 localizes to cell‐cell junctions and contributes to establishing cell polarity. PLoS One. 2015;10(6):e0129634. 26068882 10.1371/journal.pone.0129634 PMC4466050

[61] Luo W , Zhao X , Jin H , et al. Akt1 signaling coordinates BMP signaling and β‐catenin activity to regulate second heart field progenitor development. Development. 2015;142(4):732‐742. 25670795 10.1242/dev.119016

[62] Zhou L , Ercolano E , Ammoun S , Schmid MC , Barczyk MA , Hanemann CO . Merlin‐deficient human tumors show loss of contact inhibition and activation of Wnt/β‐catenin signaling linked to the PDGFR/Src and Rac/PAK pathways. Neoplasia. 2011;13(12):1101‐1112. 22247700 10.1593/neo.111060 PMC3257450

[63] Kim M , Kim S , Lee SH , et al. Merlin inhibits Wnt/beta‐catenin signaling by blocking LRP6 phosphorylation. Cell Death Differ. 2016;23(10):1638‐1647. 27285107 10.1038/cdd.2016.54 PMC5041192

[64] Rong R , Surace EI , Haipek CA , Gutmann DH , Ye K . Serine 518 phosphorylation modulates merlin intramolecular association and binding to critical effectors important for NF2 growth suppression. Oncogene. 2004;23(52):8447‐8454. 15378014 10.1038/sj.onc.1207794

[65] Wei BL , Arora VK , Raney A , et al. Activation of p21‐activated kinase 2 by human immunodeficiency virus type 1 Nef induces merlin phosphorylation. J Virol. 2005;79(23):14976‐14980. 16282498 10.1128/JVI.79.23.14976-14980.2005 PMC1287594

[66] Kong X , Gan H , Hao Y , et al. CDK11p58 phosphorylation of PAK1 Ser174 promotes DLC2 binding and roles on cell cycle progression. J Biochem. 2009;146(3):417‐427. 19520772 10.1093/jb/mvp089

[67] Grassart A , Meas‐Yedid V , Dufour A , Olivo‐Marin JC , Dautry‐Varsat A , Sauvonnet N . Pak1 phosphorylation enhances cortactin‐N‐WASP interaction in clathrin‐caveolin‐independent endocytosis. Traffic. 2010;11(8):1079‐1091. 20444238 10.1111/j.1600-0854.2010.01075.x

[68] Min JK , Park HS , Lee YB , Kim JG , Kim JI , Park JB . Cross‐talk between Wnt signaling and Src tyrosine kinase. Biomedicine. 2022;10(5):1112. 10.3390/biomedicines10051112 PMC9138253 35625853

[69] Weng J , Yu L , Chen Z , et al. β‐Catenin phosphorylation at Y654 and Y142 is crucial for high mobility group box‐1 protein‐induced pulmonary vascular hyperpermeability. J Mol Cell Cardiol. 2019;127:174‐184. 30592964 10.1016/j.yjmcc.2018.12.012

[70] Li H , Wang Y , Su R , et al. Dimethyl fumarate combined with Vemurafenib enhances anti‐melanoma efficacy via inhibiting the hippo/YAP, NRF2‐ARE, and AKT/mTOR/ERK pathways in A375 melanoma cells. Front Oncol. 2022;12:794216. 35141161 10.3389/fonc.2022.794216 PMC8820202

[71] Basu S , Totty NF , Irwin MS , Sudol M , Downward J . Akt phosphorylates the yes‐associated protein, YAP, to induce interaction with 14‐3‐3 and attenuation of p73‐mediated apoptosis. Mol Cell. 2003;11(1):11‐23. 12535517 10.1016/s1097-2765(02)00776-1

[72] Huang Z , Hu J , Pan J , et al. YAP stabilizes SMAD1 and promotes BMP2‐induced neocortical astrocytic differentiation. Development. 2016;143(13):2398‐2409. 27381227 10.1242/dev.130658 PMC4958318

[73] Martin WF , Allen JF . An algal greening of land. Cell. 2018;174(2):256‐258. 30007415 10.1016/j.cell.2018.06.034

[74] Schlegelmilch K , Mohseni M , Kirak O , et al. Yap1 acts downstream of α‐catenin to control epidermal proliferation. Cell. 2011;144(5):782‐795. 21376238 10.1016/j.cell.2011.02.031 PMC3237196

[75] Li Y , Zhou H , Li F , et al. Angiomotin binding‐induced activation of Merlin/NF2 in the Hippo pathway. Cell Res. 2015;25(7):801‐817. 26045165 10.1038/cr.2015.69 PMC4493278

[76] Xu C , Wang L , Zhang Y , et al. Tubule‐specific Mst1/2 deficiency induces CKD via YAP and non‐YAP mechanisms. J Am Soc Nephrol. 2020;31(5):946‐961. 32253273 10.1681/ASN.2019101052 PMC7217407

[77] Meng Z , Moroishi T , Mottier‐Pavie V , et al. MAP4K family kinases act in parallel to MST1/2 to activate LATS1/2 in the hippo pathway. Nat Commun. 2015;6:8357. 26437443 10.1038/ncomms9357 PMC4600732

[78] Ponce DP , Maturana JL , Cabello P , et al. Phosphorylation of AKT/PKB by CK2 is necessary for the AKT‐dependent up‐regulation of β‐catenin transcriptional activity. J Cell Physiol. 2011;226(7):1953‐1959. 21506126 10.1002/jcp.22527

