# Supplementary material for: Retinoic acid induced specific changes in the phosphoproteome of C17.2 neural stem cells
Source: J Cell Mol Med. 2024 Mar 20;28(7):e18205. doi: 10.1111/jcmm.18205 (PMC10951872; doi:10.1111/jcmm.18205)
Supplement: Supplementary file 1 — Figure S1. Figure S2. Figure S3. [file JCMM-28-e18205-s004.docx]

**Supplementary material**


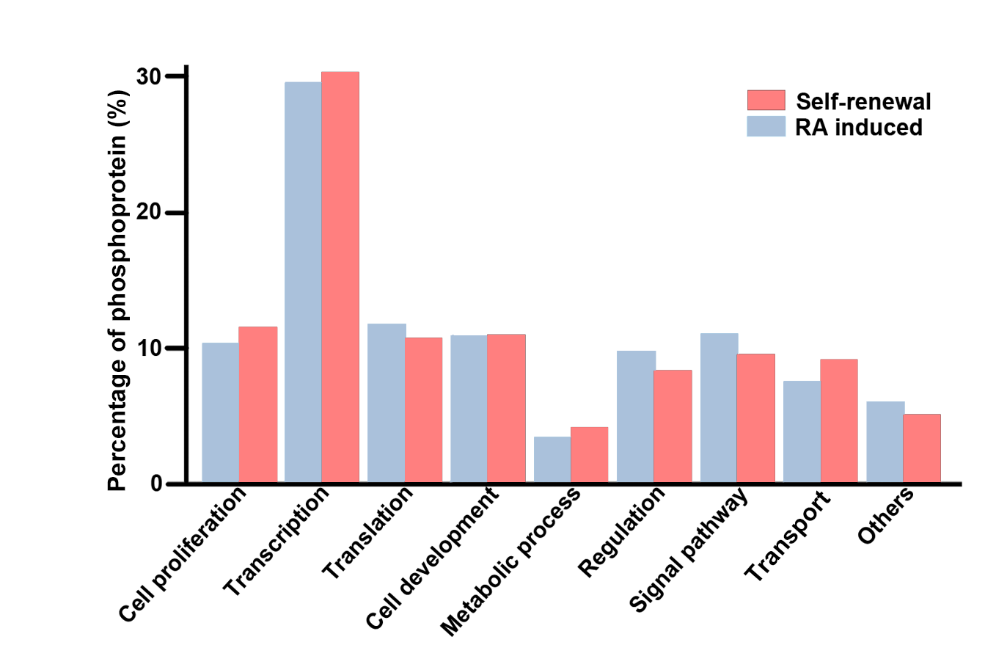


**Figure S1. Gene Ontology analysis of all proteins in self-renewal and RA-induced group.**

Proteins in self-renewal and RA-induced group were analyzed and compared by Gene Ontology. GO in molecular function were exhibited.


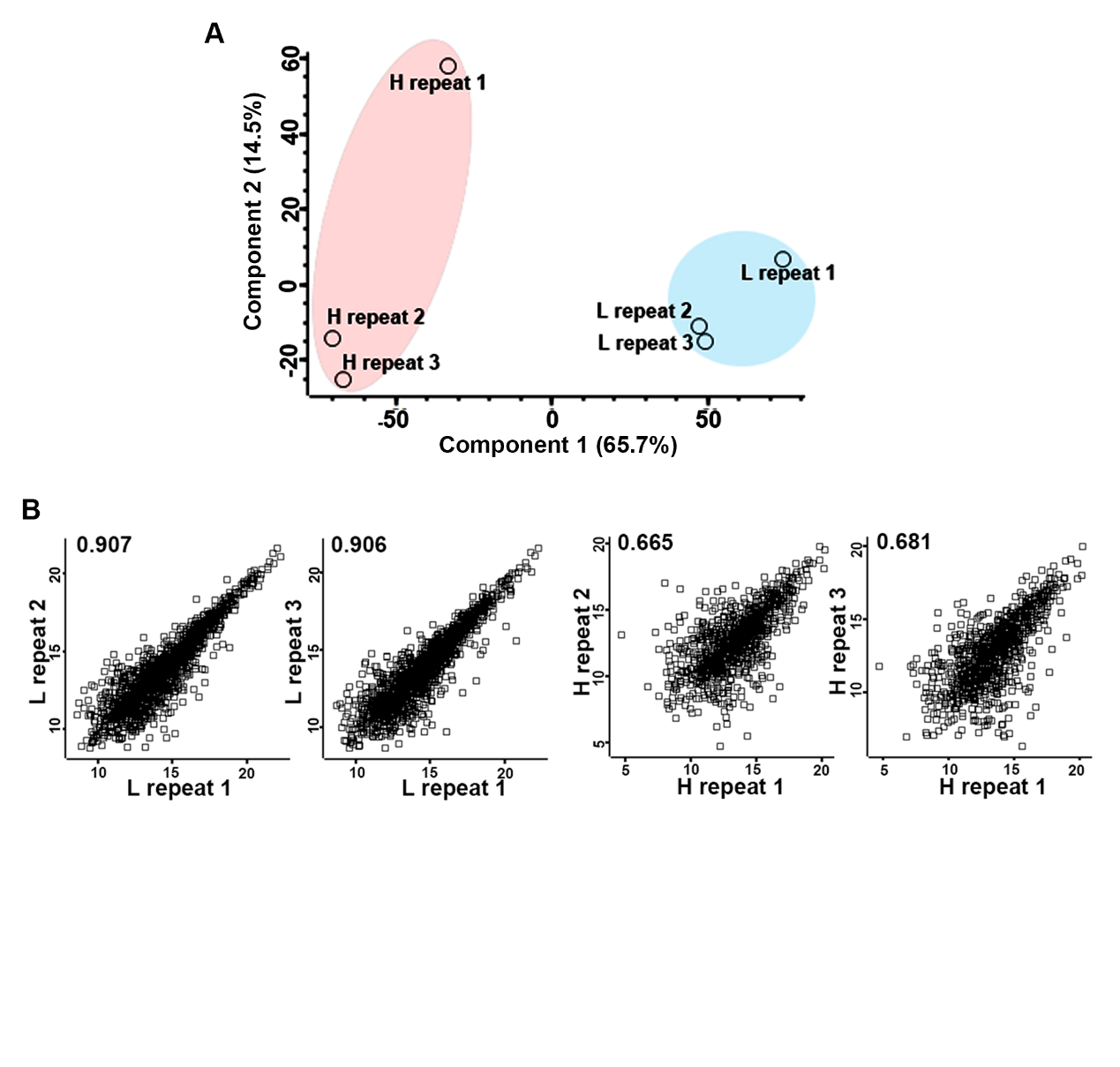


**Figure S2. Quantitative proteomic analysis of phosphopeptide in self-renewal and RA-induced group.**

(A) PCA analysis of dimethyl labeling peptides of self-renewal (L) and RA-induced (H) group in three biological replicates. (B) The correlation between each replicate of self-renewal (L) or RA-induced (H).


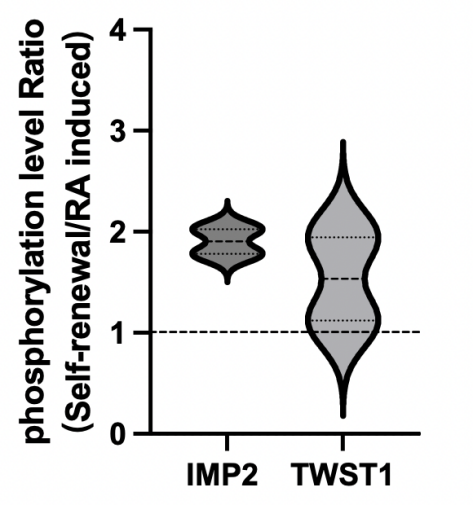


**Figure S3. Differentially phosphorylation level of cell cycle related genes IMP2 and** **Twist1 in self-renewal and RA-induced group.**

Phosphorylation level ratio of IMP2 and Twist1 were exhibited by violin plot.

**Table S1. Phosphorylation sites were identified in self-renewal group and RA-induced group.**

**Table S2. Differentially expressed phosphoproteins were identified in self-renewal or RA-induced group.**

**Table S3. Quantified unique phosphoproteins were identified in quantitative phosphoproteomics**
